# Signatures of Co-Deregulated Genes and Their Transcriptional Regulators in Kidney Cancers

**DOI:** 10.3390/ijms24076577

**Published:** 2023-03-31

**Authors:** Ioanna Ioannou, Angeliki Chatziantoniou, Constantinos Drenios, Panayiota Christodoulou, Malamati Kourti, Apostolos Zaravinos

**Affiliations:** 1 Department of Life Sciences, School of Sciences, European University Cyprus, Nicosia 2404, Cyprus; 2 Cancer Genetics, Genomics and Systems Biology Group, Basic and Translational Cancer Research Center (BTCRC), Nicosia 1516, Cyprus; 3 School of Medicine, European University Cyprus, Nicosia 2404, Cyprus; 4 Angiogenesis and Cancer Drug Discovery Group, Basic and Translational Cancer Research Center (BTCRC), Nicosia 1516, Cyprus

**Keywords:** kidney cancer, KIRC, KIRP, KICH, tumor heterogeneity, Characteristic Direction, co-deregulated genes, single-gene perturbation, single-drug perturbation, drug repurposing, GEO2Enrichr, X2K

## Abstract

There are several studies on the deregulated gene expression profiles in kidney cancer, with varying results depending on the tumor histology and other parameters. None of these, however, have identified the networks that the co-deregulated genes (co-DEGs), across different studies, create. Here, we reanalyzed 10 Gene Expression Omnibus (GEO) studies to detect and annotate co-deregulated signatures across different subtypes of kidney cancer or in single-gene perturbation experiments in kidney cancer cells and/or tissue. Using a systems biology approach, we aimed to decipher the networks they form along with their upstream regulators. Differential expression and upstream regulators, including transcription factors [MYC proto-oncogene (MYC), CCAAT enhancer binding protein delta (CEBPD), RELA proto-oncogene, NF-kB subunit (RELA), zinc finger MIZ-type containing 1 (ZMIZ1), negative elongation factor complex member E (NELFE) and Kruppel-like factor 4 (KLF4)] and protein kinases [Casein kinase 2 alpha 1 (CSNK2A1), mitogen-activated protein kinases 1 (MAPK1) and 14 (MAPK14), Sirtuin 1 (SIRT1), Cyclin dependent kinases 1 (CDK1) and 4 (CDK4), Homeodomain interacting protein kinase 2 (HIPK2) and Extracellular signal-regulated kinases 1 and 2 (ERK1/2)], were computed using the Characteristic Direction, as well as GEO2Enrichr and X2K, respectively, and further subjected to GO and KEGG pathways enrichment analyses. Furthermore, using CMap, DrugMatrix and the LINCS L1000 chemical perturbation databases, we highlight putative repurposing drugs, including Etoposide, Haloperidol, BW-B70C, Triamterene, Chlorphenesin, BRD-K79459005 and β-Estradiol 3-benzoate, among others, that may reverse the expression of the identified co-DEGs in kidney cancers. Of these, the cytotoxic effects of Etoposide, Catecholamine, Cyclosporin A, BW-B70C and Lasalocid sodium were validated in vitro. Overall, we identified critical co-DEGs across different subtypes in kidney cancer, and our results provide an innovative framework for their potential use in the future.

## 1. Introduction

Kidney cancer accounts for 2% of all cancers worldwide and is the third most common type of urinary tract cancer, killing over 131,000 people each year [1]. Clear-cell renal cell carcinoma (ccRCC or KIRC) is the most common type of kidney cancer [2], which is best described by the loss of the chr3p locus [3,4], which contains the Von Hippel–Lindau tumor suppressor (VHL) gene. VHL is linked to key metabolic pathways in renal cancer [5] as well as other tumor suppressor genes such as BRCA1-associated protein 1 (BAP1) [6,7], which is required for normal kidney development and function. Papillary renal cell carcinoma (papRCC or KIRP) and chromophobe renal cell carcinoma (chroRCC or KICH) (10–15% and 5% of total renal tumors, respectively) follow ccRCC.

Several studies aim to find differentially expressed genes (DEGs) between kidney cancer and normal tissue without considering that the dysregulation patterns occur at multiple gene expression levels and can differ across heterogeneous kidney cancer subtypes. DEGs vary from study to study depending on tumor histology, molecular subtype, tumor stage/grade and/or other biological parameters or computational methodology. To this extent, the analysis of genes subjected to simultaneous deregulation alongside their commonly altered signaling pathways could reveal the broad pathogenetic model of kidney carcinogenesis and further aid in identifying treatment(s) for kidney tumors with diverse molecular profiles.

A new multivariate method used to measure deregulated gene expression signatures is the Characteristic Direction (CD), which uses the orientation of the separating hyperplane from a linear classification scheme to define a direction that characterizes differential expression [8].

Here, we hypothesized that specific hub proteins [transcription factors (TFs) and kinases] are the regulatory players in gene expression of co-DEGs in kidney cancer. In fact, by searching large datasets of differentially expressed genes, those modulatory links can be better understood. To investigate this further, we extracted gene expression signatures from 10 Gene Expression Omnibus (GEO) datasets and constructed the co-deregulated gene networks consisting of their upstream transcriptional regulators, such as transcription factors and kinases, and the hubs in these networks. Furthermore, we identified and evaluated in vitro the reversible effect of repurposing drugs on the expression of the proposed co-DEGs. The lists of co-deregulated gene signatures that we propose corroborate previous findings and provide new insights into the development and treatment of kidney cancer(s).

## 2. Results

We analyzed the co-DEGs across 10 independent studies using a systems biology approach and classified them into two groups: (1) renal cancer vs. the normal tissue and (2) single-gene perturbation vs. the wild-type cells or tissue. We then filtered and removed genes that were found to be deregulated in a single study and concluded the co-DEGs for each of the above-mentioned groups. In specific, we found 72 co-upregulated and 74 co-downregulated genes in renal cancer(s) vs. normal renal studies, as well as 89 co-upregulated and 23 co-downregulated genes in single-gene perturbation studies. We discovered theTFs, protein–protein interactions (PPIs) and kinases driving the observed changes in gene expression. We identified the phosphorylation reactions that might be carried out by upstream regulators in particular kinases, acting as regulators of gene expression in each category. The top 10 TFs and protein kinases from both groups of co-DEGs were categorized based on the highest value of a combined score between the *p*-value and the z-score. We also found the top GO- and KEGG-enriched terms for the co-DEGs and investigated the drugs that repress or increase their expression using the DrugMatrix, cMap and LINCS L1000 databases. The effects of five drugs (BW-B70C, Lasalocid sodium, Catecholamine, Cyclosporin A and Etoposide) were validated in vitro.

### 2.1. Co-Deregulated Genes in Kidney Cancers vs. the Normal Tissue

**Figure 1:**
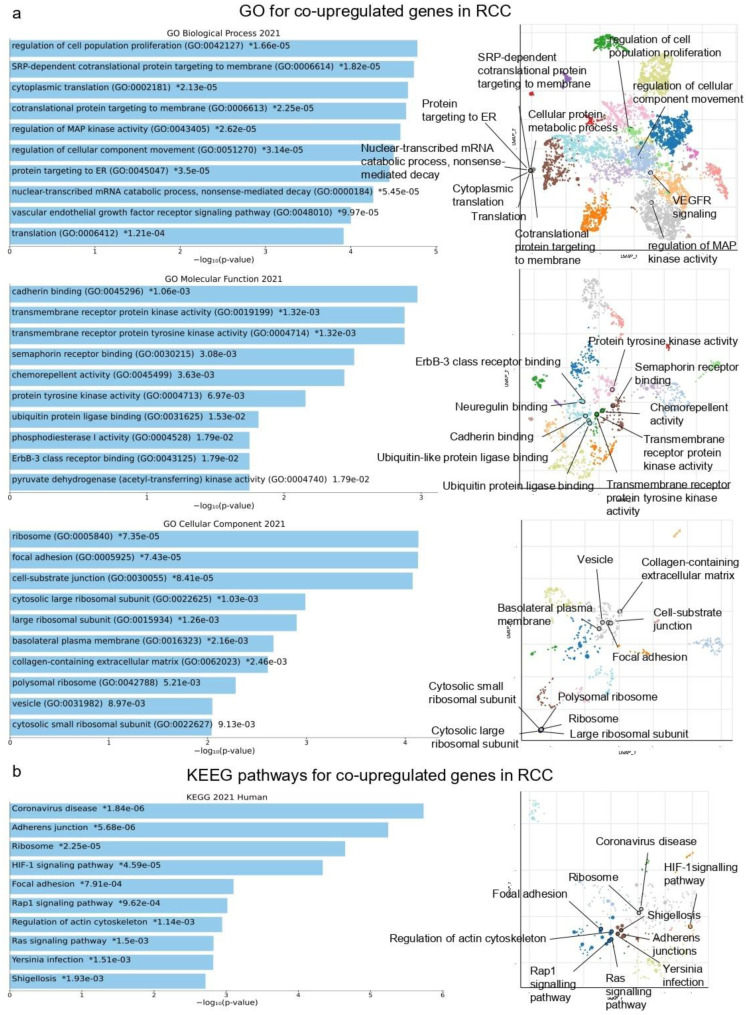
The bar charts (left) depict the top 10 enriched Gene Ontology (GO) terms (**a**) and KEGG pathways (**b**) in which the top 250 co-upregulated genes in RCC participate, along with their corresponding *p*-values. Asterisks (*) indicate the terms with significant adjusted *p*-values (<0.05). The scatterplots (right) were created using UMAP and depict clusters of similar gene sets. The significantly enriched terms of the associated gene sets are denoted.

We looked at four different GEO datasets to see which co-DEGs were present in renal cancer tissues compared to their normal tissue. The co-upregulated genes in this category were substantially enriched “regulation of cell population proliferation”, “SRP-dependent co-translational protein targeting to membrane” and “cytoplasmic translation” (GO Biological Process); “cadherin binding”, “Transmembrane receptor protein kinase activity” and “Semaphorin receptor binding” (GO Molecular Function); and “ribosome”, “focal adhesion” and “cell-substrate junction” (GO Cellular Component) (Figure 1a).

We also discovered the top 10 KEGG pathways of the commonly up-regulated and down-regulated genes. The co-upregulated genes in RCC are mostly related to the pathways “Coronavirus disease”, “Adherens junction”, “Ribosome”, “HIF-1 signaling pathway”, “Focal adhesion”, “Rap1 signaling pathway”, “Regulation of actin cytoskeleton”, “Ras signaling pathway”, “Yersinia infection” and “Shigellosis” (Figure 1b and Table S1).

**Figure 2:**
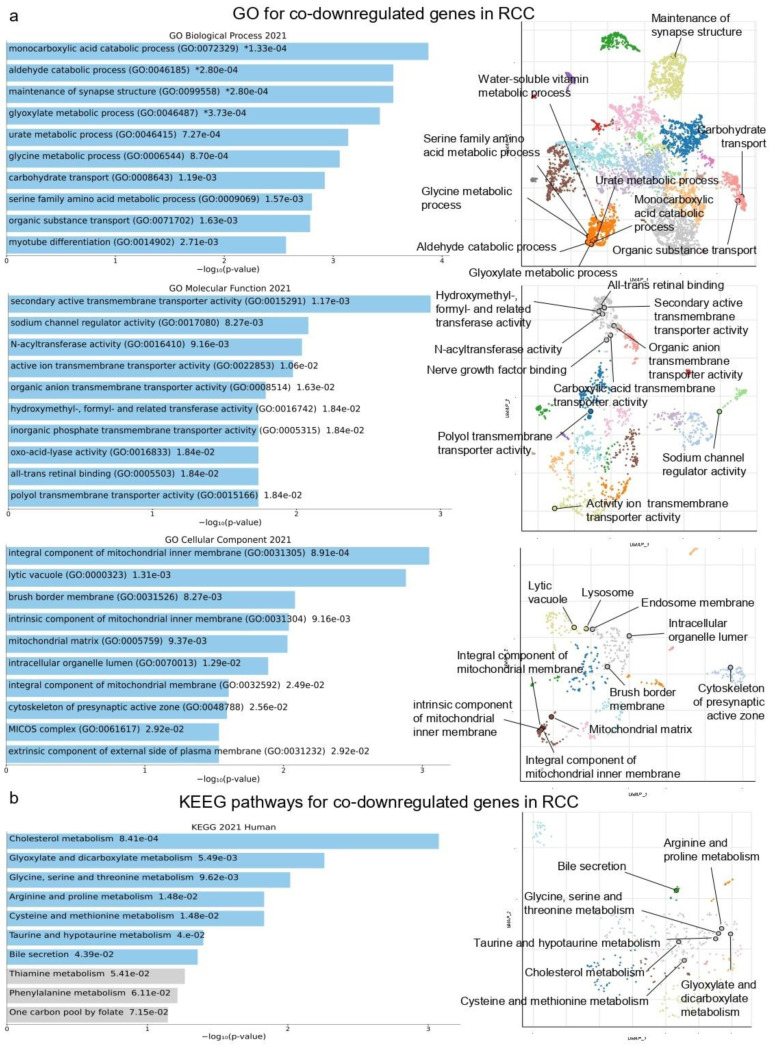
The bar charts (left) depict the top 10 enriched Gene Ontology (GO) terms (**a**) and KEGG pathways (**b**) in which the top 250 co-downregulated genes in RCC participate, along with their corresponding *p*-values. Asterisks (*) indicate the terms with significant adjusted *p*-values (<0.05). The scatterplots (right) were created using UMAP and depict clusters of similar gene sets. The significantly enriched terms of the associated gene sets (also highlighted in blue color in the bar charts) are denoted. The GO and KEGG terms appearing in grey color in the bar charts, are non-significant.

Furthermore, the co-downregulated genes were enriched in “monocarboxylic acid catabolic process”, “aldehyde catabolic process” and “maintenance of synapse structure” (GO Biological Process); “secondary active transmembrane transporter activity” “sodium channel regulator activity” and “N-acyltransferase activity” (GO Molecular function); and “integral component of mitochondrial inner membrane”, “lytic vacuole” and “brush border membrane” (GO Cellular Component) (Figure 2a).

The co-downregulated genes, on the other hand, were enriched in the “Cholesterol metabolism”, “Glyoxylate and dicarboxylate metabolism”, “Glycine”, “Serine and threonine metabolism”, “Arginine and proline metabolism”, “Cysteine and methionine metabolism”, “Taurine and hypotaurine metabolism” and “Bile secretion” pathways (Figure 2b and Table S1).

**Figure 3:**
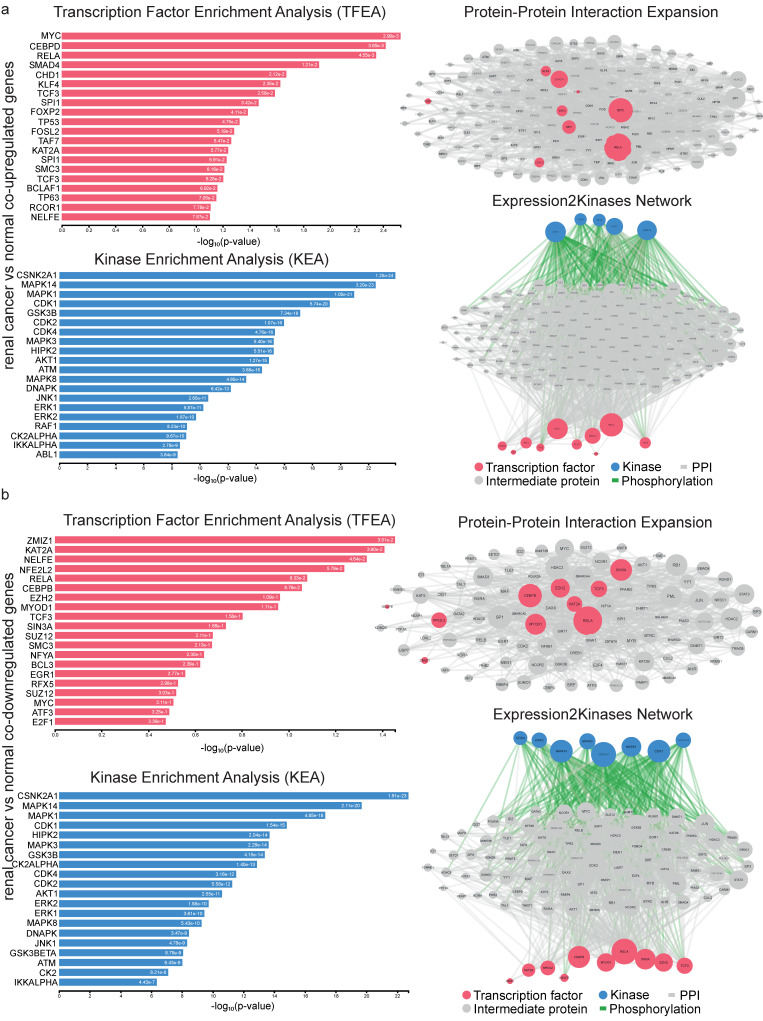
Upstream regulatory networks for co-upregulated (**a**) and co-downregulated (**b**) gene signatures in kidney cancers vs. the normal tissue. The networks depict transcription factors (TFs, red nodes), intermediate proteins (grey nodes) and kinases (blue nodes). Grey edges indicate PPI interactions and green edges depict kinase-driven phosphorylation events. Node size is relative to the levels of expression degree. Upstream regulatory networks were constructed using the eXpression2Kinases (X2K) algorithm.

We then built a PPI sub-network using eXpression2Kinases (X2K) and the proteins encoded by the co-DEGs, and we ran a topological analysis on it to identify the hub proteins. Among the co-upregulated genes, we discovered 40 hub proteins, including the TFs MYC, CEBPD, RELA, SMAD4 (Mothers against decapentaplegic homolog 4) and CHD1 (Chromodomain-helicase DNA-binding 1), and the kinases CSNK2A1, MAPK14, MAPK1, CDK1 and GSK3B (Glycogen synthase kinase-3 beta), among others (Figure 3a and Table S2). These hub proteins are important for the development of renal cancer, and MYC was the most common hub, being upregulated in both categories.

Among the co-downregulated genes, we highlighted the following TFs: ZMIZ1, KAR2A (KAR2 Hsp70 family ATPase), NELFE, NFE2L2 (NFE2 like bZIP transcription factor 2), RELA, CEBPB, EZH2, MYOD1 and TCF3 (Transcription factor 3), and the following kinases: CSNK2A1, MAPK14, MAPK1, CDK1, HIPK2 and GSK3B, among others (Table S2).

Interestingly, Casein kinase 2 alpha 1 (CSNK2A1), Mitogen-Activated Protein Kinase 14 (MAPK14) and Mitogen-Activated Protein Kinase 1 (MAPK1) were significantly ranked in both categories (co-up- and co-downregulated genes) according to the kinase enrichment analysis (KEA) (Figure 3b).

### 2.2. Co-Deregulated Genes within Single-Gene Perturbation Studies in Kidney Cancers

**Figure 4:**
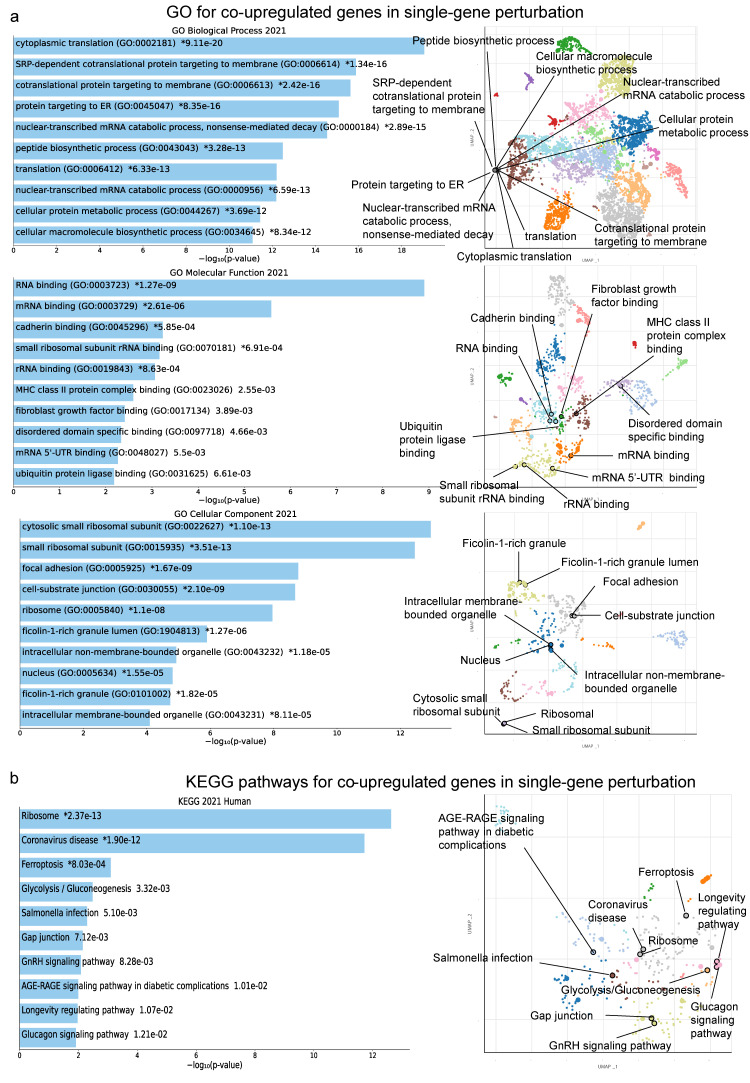
The bar charts (left) depict the top 10 enriched Gene Ontology (GO) terms (**a**) and the KEGG pathways (**b**) for the top 250 co-upregulated genes in kidney cancer cells with a single-gene perturbation, along with their corresponding *p*-values. Asterisks (*) indicate the terms with significant adjusted *p*-values (<0.05). The scatterplots (right) were created using UMAP and depict clusters of similar gene sets. The significantly enriched terms of the associated gene sets are denoted.

The co-upregulated genes in kidney cancer-related single-gene perturbation studies were enriched in “cytoplasmic translation”, “SRP-dependent co-translational protein targeting to membrane” and “co-translational protein targeting to membrane” (GO Biological Process); “RNA binding”, “mRNA binding” and “cadherin binding” (GO Molecular Function); and “cytosolic small ribosomal subunit”, “small ribosomal subunit” and “focal adhesion” (GO Cellular Component) (Figure 4a). We also discovered the top 10 KEGG pathways of the co-DEGs across single-gene perturbation experiments. These were mostly enriched in “Ribosome”, “Coronavirus disease”, “Ferroptosis”, “Glycolysis/Gluconeogenesis”, “Salmonella infection”, “Gap junction”, “GnRH signaling pathway”, “AGE-RAGE signaling pathway in diabetic complications”, “Longevity regulating pathway” and “Glucagon signaling pathway” (Figure 4b).

**Figure 5:**
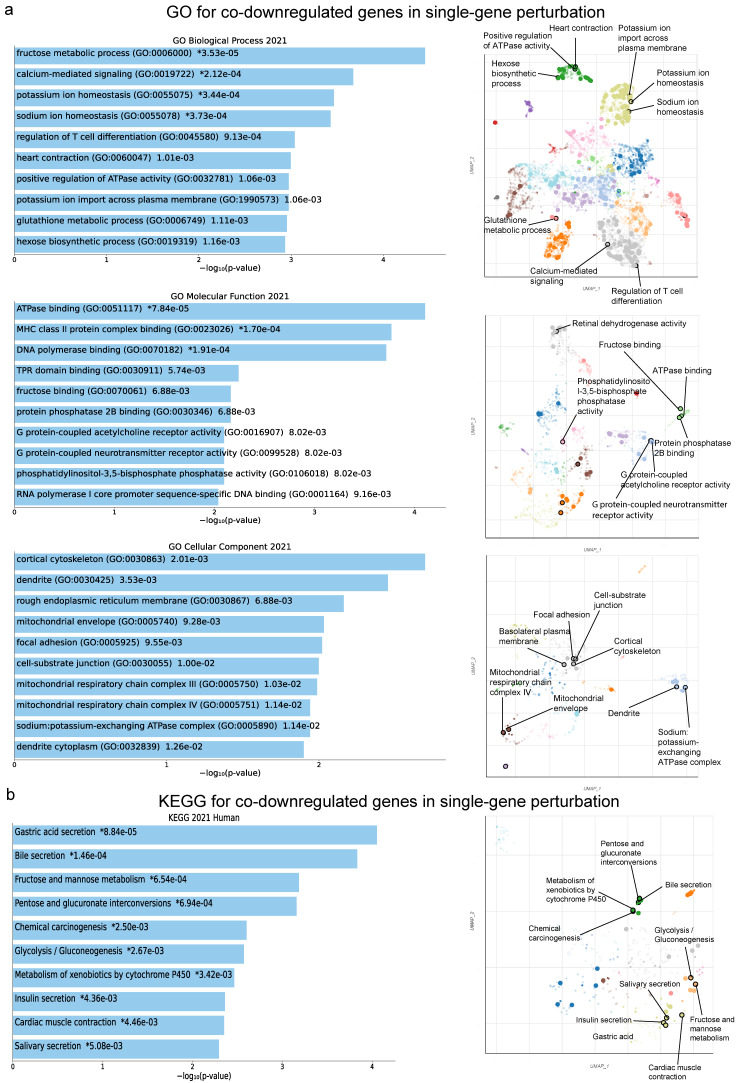
The bar charts (left) depict the top 10 enriched Gene Ontology (GO) terms (**a**) and the KEGG pathways (**b**) for the top 250 co-downregulated genes in RCC with a single-gene perturbation, along with their corresponding *p*-values. Asterisks (*) indicate the terms with significant adjusted *p*-values (<0.05). The scatterplots (right) were created using UMAP and depict clusters of similar gene sets. The significantly enriched terms of the associated gene sets are denoted.

In addition, the co-downregulated genes in this category were mainly enriched in “fructose metabolic process”, “calcium-mediated signaling” and “potassium ion homeostasis” (GO Biological Process); as well as in “ATPase binding”, “MHC class II protein complex binding” and “DNA polymerase binding” (GO Molecular Function); and “cortical cytoskeleton”, “dendrite” and “rough endoplasmic reticulum membrane” (GO Cellular Component) (Figure 5a). In addition, the co-downregulated genes in this category were enriched in “Gastric acid secretion”, “Bile secretion”, “Fructose and mannose metabolism”, “Pentose and glucuronate interconversions”, “Chemical carcinogenesis”, “Glycolysis/Gluconeogenesis”, “Metabolism of xenobiotics by cytochrome P450”, “Insulin secretion”, “Cardiac muscle contraction” and “Salivary secretion” (Figure 5b).

**Figure 6:**
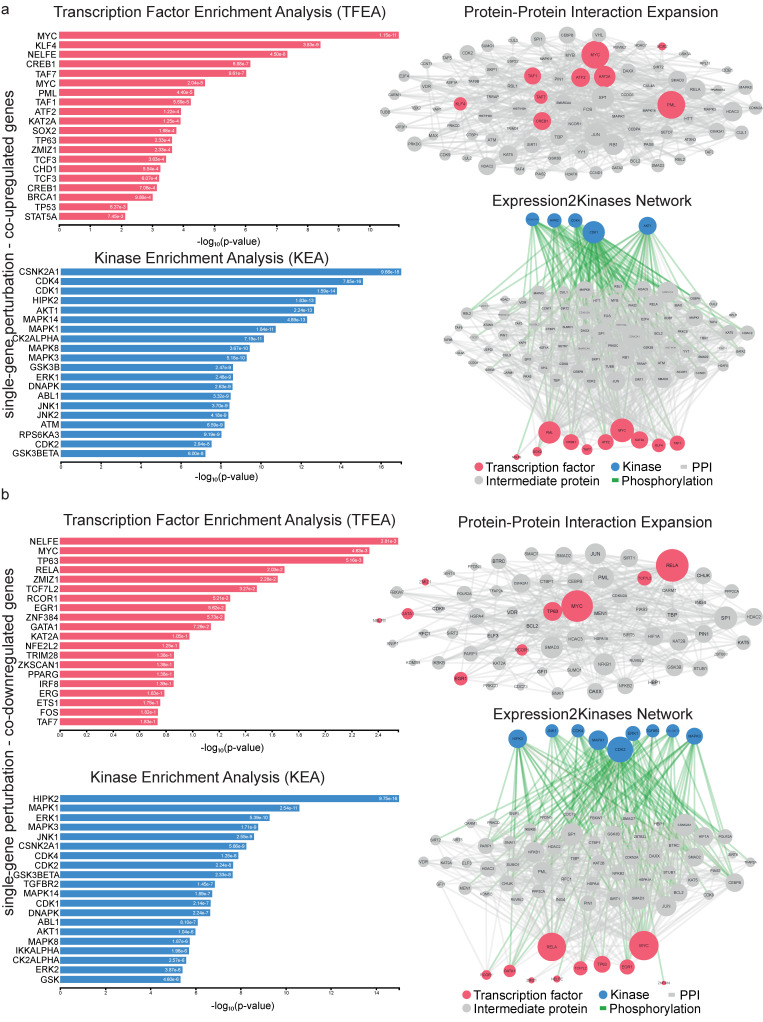
Upstream regulatory networks for co-upregulated (**a**) and co-downregulated (**b**) gene signatures in single-gene perturbation experiments in kidney cancers vs. the normal tissue. The networks depict transcription factors (TFs, red nodes), intermediate proteins (grey nodes) and kinases (blue nodes). Grey edges indicate PPI interactions and green edges depict kinase-driven phosphorylation events. Node size is relative to expression. Upstream regulatory networks were constructed using the eXpression2Kinases (X2K) algorithm.

Following, we built a PPI sub-network composed of the TFs and protein kinases that drive the co-deregulated genes in single-gene experiments in RCC. We prioritized the TFs MYC, KLF4, NELFE, CREB1, TAF7, ATF2 and KAT2A, as these were among the most prominent hubs across the co-upregulated genes. Similarly, the kinases CSNK2A1, CDK4, CDK1, HIPK2, AKT1, MAPK14 and MAPK1 were hubs for the co-upregulated genes (Figure 6a). NELFE, on the other hand, was the most prominent hub TF, along with MYC, TP63 RELA, ZMIZ1, TCF7L2 and RCOR1; whereas CSNK2A1, CDK4, CDK1, HIPK2, AKT1, MAPK14 and MAPK14 (Figure 6b) were the top enriched protein kinases for the co-downregulated genes in this category.

### 2.3. Validation of the Co-Deregulated Gene Signatures in the TCGA Database

**Figure 7:**
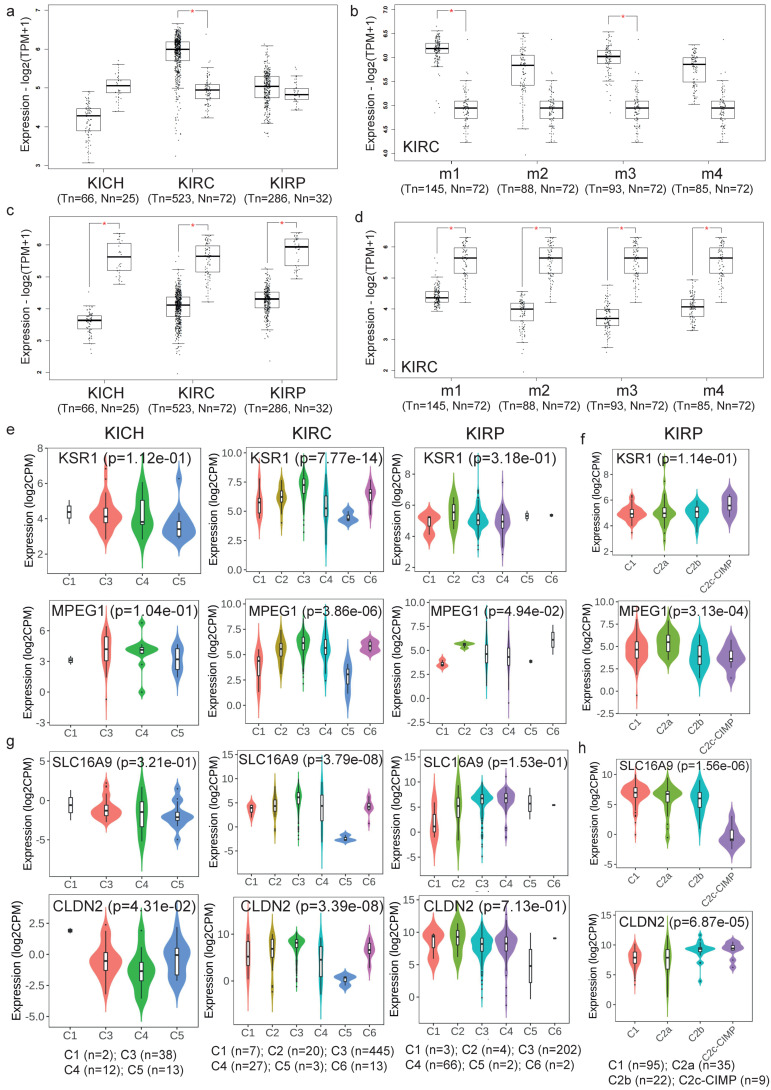
Validation of the co-UP (**a**) and co-DOWN (**c**) gene signatures in the TCGA-KICH, TCGA-KIRC and TCGA-KIRP datasets, as well as in four mRNA clusters in KIRC (**b**,**d**). Results with a |log_2_FC ≥ 1| and *p*-value < 0.01 were considered statistically significant (*). The expression of KSR1 and MPEG1, two genes within the co-UP signature, were validated across different immune (**e**) and molecular (**f**) subtypes in kidney cancer. The expression of SLC16A9 and CLDN2, two genes within the co-DOWN signature, were also validated across different immune (**g**) and molecular (**h**) subtypes in kidney cancer.

Following, we validated the calculated expression signatures of the co-UP or co-DOWN-regulated gene signatures across the 3 kidney cancer subtypes (KICH, 66 tumors and 25 normal samples; KIRC, 523 tumors and 72 normal samples; and KIRP, 286 tumors and 32 normal samples), as well as across 4 mRNA clusters in KIRC. Apart from the co-UP signature in KICH, the rest of the comparisons validated the results from the CD algorithm (Figure 7a–d).

We also explored the expression of specific genes from the co-UP and co-DOWN signatures across different molecular and immune subtypes in kidney cancer. For example, from the co-UP gene signature, we found that the Kinase suppressor of ras 1 (KSR1 or RSU2) and Macrophage expressed 1 (MPEG1) are expressed higher in the C3 (inflammatory) immune subtype of KICH and KIRC and in the C2 (IFNγ-dominant) immune subtype of KIRP (Figure 7e). In addition, KSR1 expression was higher in the C2c- CpG island methylator phenotype (CIMP) molecular subtype of KIRP tumors, whereas MPEG1 expressed higher in the C2a molecular subtype of KIRP (Figure 7f). KSR1 promotes the phosphorylation of Raf family members and the activation of downstream MAPK1 and/or MAPK3 kinases, both in response to EGF and to cAMP [9]. These findings support the existence of a correlation between MPEG1 and anti-tumor immunity.

Two more genes from the list of co-DOWN signatures that we further explored are Solute carrier family 16, member 9 (SLC16A9) and Claudin 2 (CLDN2). SLC16A9 is found in the cell membrane and acts as a proton-linked monocarboxylate transporter. CDLN2, on the other hand, plays a major role in tight junction-specific obliteration of the intercellular space through calcium-independent cell-adhesion activity. SLC16A9 was highly expressed in the C3 (inflammatory) and C4 (lymphocyte depleted) immune subtypes of all three different subtypes of kidney tumors (KICH, KIRC and KIRP) (Figure 7g), and it was lowly expressed in the C2c-CIMP molecular subtype of KIRP (Figure 7h). CLDN2, on the other hand, was upregulated in the C3 (inflammatory) and C5 (immunologically quiet) immune subtypes in KICH tumors and in the C1 (wound healing), C2 (IFN-gamma dominant) and C3 (inflammatory) immune subtypes in KIRC and KIRP tumors (Figure 7g). In addition, CDLN2 expression was higher in the C2b and C2c-CIMP molecular subtypes in KIRP (Figure 7h).

### 2.4. Gene Set Cancer Analysis

Next, we run gene set variation analysis to estimate the integrated level of expression in three TCGA datasets concerning different subtypes of kidney cancer (TCGA-KIRP, TCGA-KICH and TCGA-KIRC). The gene set that we used was composed of the previously found hub genes (MYC, CEBPD, RELA, ZMIZ1, KAT2A, KLF4, NELFE, MAPK14, MAPK1, CSNK2A1, CDK4, CDK1, HIPK2 and ERK1). This analysis verified our previous findings. For example, MYC, CDK1 and CDK4 were found to be significantly up-regulated in KIRC and KIRP; whereas CSNK2A1 was significantly up-regulated in KICH.

**Figure 8:**
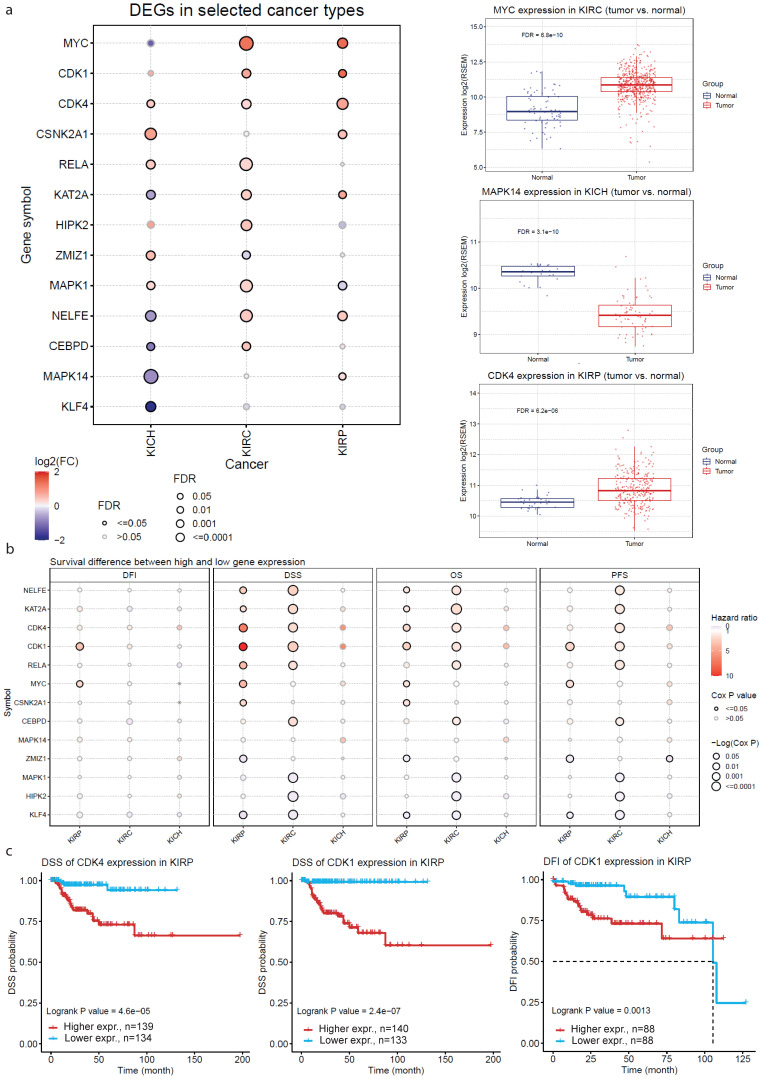
(**a**) Analysis of the differential expression between different kidney tumors in the TCGA dataset (KIRP, KIRC and KICH) and their adjacent normal samples. The bubble colors from purple to red represent the fold change between kidney tumor and normal samples. The size of each dot correlates the significance of FDR. The dots were filtered by the fold change (FC > 2) and statistical significance (FDR ≤ 0.05). Detailed expression of MYC, MAPK14 and CDK4 in KIRC, KICH and KIRP, respectively, are shown to the right. (**b**) Estimation of patient survival differences (OS, PFS, DSS and DFI) between high and low gene expression groups. The colors from blue to red represent the hazard ratio (HR) and size represents statistical significance in the bubble plot. The black outline border indicates Cox *p* ≤ 0.05. (**c**) Exemplary associations of high CDK4 and CDK1 with worse prognosis in KIRP are depicted in the Kaplan–Meier curves.

In addition, we estimated the survival (overall survival (OS); progression-free survival (PFS); disease-specific survival (DSS); and disease-free interval (DFI)) difference between high and low gene expression groups, and we found significant associations between high CDK1 and CDK4 expression and DSS in KIRP. We also found significant association between NELFE, KAT2A, CDK4, CDK1, RELA, MAPK1, HIPK2 and KLF4 and OS (as well as PFS) in KIRC (Figure 8).

**Figure 9:**
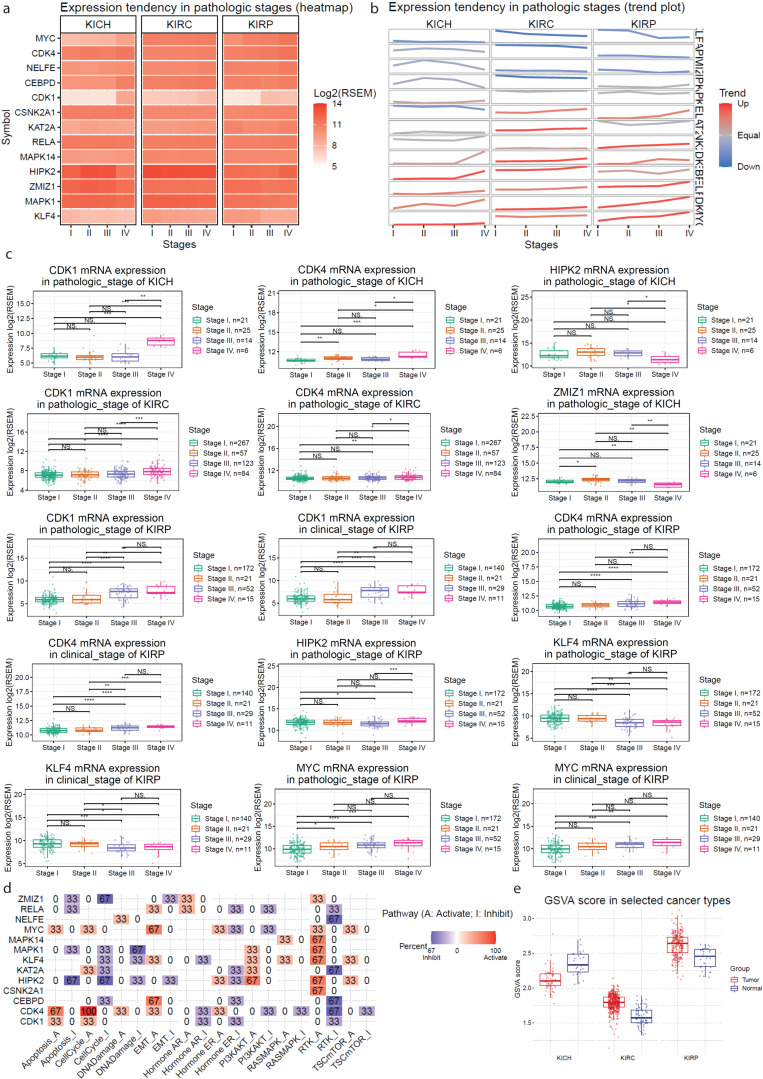
The heatmap (**a**) and trend plot (**b**) present the gene mRNA expression profile from stage I to stage IV of kidney cancers (KICH, KIRC and KIRP) in the TCGA database. The trend line colors from blue to red represents the tendency from fall to rise. The *p*-values were calculated using the Mann–Kendall test for trend analysis. *p*-values < 0.05 (*), <0.01 (**), <0.001 (***) or <0.0001 (****) were considered statistically significant, else if *p*-values were >0.05, were considered non-significant (NS). (**c**) Examples of the increased or decreased mRNA expression of various genes in pathologic and clinical stages of KICH, KIRC and KIRP. The Wilcoxon or ANOVA tests were used to assess statistical significance between 2 or >2 stage groups, respectively. *p*-values < 0.05 were considered statistically significant. NS, non-significant. (**d**) The percentage of cancers in which the mRNA expression of the genes of interest has a potential effect on pathway activity. Red color, activatory (A) effect; blue color, inhibitory (I) effect. The number in each cell indicates the percentage of cancer types in which each gene shows significant association with a specific pathway, among the three kidney tumor subtypes. (**e**) The box plots compare the GSVA scores between kidney cancer (KIRCH, KIRC and KIRP) and normal samples. GSVA scores represent the variation of gene set activity over a specific cancer sample population in an unsupervised manner, which was calculated through the GSVA R package. Briefly, the GSVA score represents the integrated level of the expression of the gene set, which is positively correlated with gene expression.

We also estimated the mRNA expression profiles of the key hub genes across different stages in KICH, KIRC and KIRP. Keeping in mind that these profiles represent a snapshot of the underlying cancer process, we observed that the expression of CDK1 and CDK4 increased significantly with the tumor’s pathologic stage in KICH (CDK1, *p* = 1.37 × 10^−5^ and CDK4, *p* = 6.39 × 10^−3^). Similar trends were also noticed for CEBPD (*p* = 0.02), KAT2A (*p* = 0.03), KLF4 (*p* = 5.74 × 10^−6^), NELFE (*p* = 9.68 × 10^−3^) and RELA (*p* = 6.80 × 10^−3^) in KICH, whereas HIPK2 (*p* = 0.01), ZMIZ2 (*p* = 3.18 × 10^−3^) levels significantly decreased in stage IV KICH tumors. In KIRC, we noticed an increased trend for CDK1, CDK4, CEBPD, HIPK2, KAT2A and KLF4 in tumors of higher pathologic stage. On the other hand, in KIRP, CDK1, CDK4, HIPK2 and MYC expression increased with the pathological and clinical stage of the tumor, whereas KLF4 decreased (Figure 9a–c).

In addition, we explored the pathway activity between high and low mRNA expression for the above-designated gene set. We found various inhibitory or activating effects for most of the genes across different pathways. For example, our results corroborate that CDK4 activates the CellCycle_A pathway (100%), but we also found a high percentage of activation (67%) from MYC and CDK4 of the EMT_A pathway. In addition, our data show a significant (67%) inhibitory effect of NELFE, KAT2A, CEBPD and CDK4 for the RTK_I pathway but also a high activation effect of HIPK2 (67%) for the PI3KAKT_A pathway (Figure 9d).

Furthermore, we calculated the GSVA scores for the designated gene list in KICH, KIRC and KIRP tumors and found that these were significantly higher in KIRC (*p* = 3.78 × 10^−21^) and KIRP (*p* = 4.57 × 10^−8^) tumors, but lower in KICH (*p* = 3.64 × 10^−7^), compared to their adjacent normal kidney tissues (Figure 9e).

### 2.5. Repurposed Drugs in Kidney Cancers

**Figure 10:**
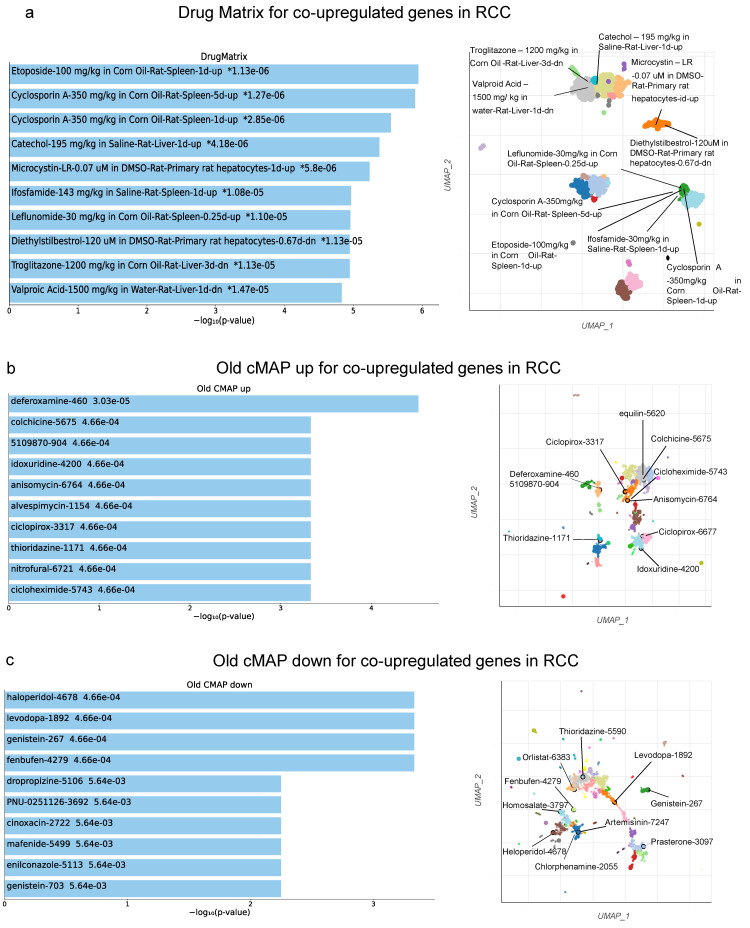
Top 10 enriched compounds that can be used as repurposing drugs against the top 250 co-upregulated genes in RCC according to DrugMatrix (**a**) and cMap (**b**,**c**) databases. The repurposed drugs upregulating or downregulating the co-upregulated genes in RCC, according to Old cMAP analysis are depicted in (**b**,**c**), respectively. Asterisks (*) indicate the terms with significant adjusted *p*-values (<0.05). The scatterplots (right) were created using UMAP and depict clusters of similar compounds. The significantly enriched terms of the associated gene sets are denoted.

Drug repurposing is an approach for identifying new uses for approved or investigational drugs that are outside the scope of the original medical indication. To identify repurposed drugs that could be used against RCC, we explored the co-DEGs using three different databases: (1) DrugMatrix, (2) cMap and (3) LINCS L1000. We then gathered the top 10 compounds from each database targeting the co-upregulated genes with the highest p-value. In specific, we found “Etoposide”, “Cyclosporin A (5 days up)”, “Cyclosporin (1 day up)”, “Catechol”, “Microcystin-LR”, “Ifosfamide”, “Leflunomide”, “Diethystilbestrol”, “Troglitazone” and “Valproic Acid” (DrugMatrix); “haloperidol”, “levodopa”, “genistein”, “fenbufen”, “dropropizine”, “PNU-0251126”, “cinoxacin”, “mafenide”, “enilconazole” and “genistein” (cMap); as well as “BW-B70C”, “BI-2536”, “XMD16-144”, “lasalocid sodium”, “brd-k19624190”, “brd-u25771771”, “brd-k15563106”, “perphenazine”, “pf 431396” and ”brd-k6462496” (LINCS L1000 Chem pert) (Figure 10).

On the other hand, the 10 compounds targeting the co-downregulated genes were as follows: “Triamterene”, “Naproxen”, “Sulfadimethoxine”, “Aspirin”, “Catechol”, “Phenacemide”, “Mefenamic Acid”, “Cytarabine”, “Foscarnet” and “Oxaliptain” (DrugMatrix); “chlorphenesin”, “pentetic acid” and “phenylpropanolamine” (cMap); and “BRD-K79459005”, “radicicol”, “AT 7519”, “mk 212”, “tanespimycin”, “ei-346”, “BRD-K96704748”, “idarubicin hcl” and “NVP-AUY922 (Luminespib)” (LINCS L1000).

### 2.6. Repurposed Drugs in Single-Gene Perturbation Experiments in Kidney Cancers

We also explored the co-DEGs across single-gene perturbation experiments in kidney cancer for repurposing drugs. The top compounds predicted to target the co-upregulated genes in this category, were as follows: “Beta-Estradiol 3-Benzoate (1 day up)”, “Gemcitabine”, “Isoprenaline”, “Rapamycin”, “Beta-Estradiol 3-Benzoate (3 days up)”, “N-Nitrosodiethylamine”, “Harringtonine”, “Methotrexate”, “4-Nitrobenzoic Acid” and “Dobutamine” (DrugMatrix); “dequalinium chloride”, “fulvestrant”, “troglitazone”, “rimexolone”, “demecolcine”, “LY-294002-5233”, “LY-294002-1007”, “phensuximide” and “testosterone” (cMap); as well as “AS-605240”, “saracatinib”, “WZ-4002”, “radicicol”, “trametinib”, “Alvocidib”, “CHIR-99021”, “AKT-inhibitor-1-2-10”, “CGP-60474” and “PD-184352” (LINCS L1000).

**Figure 11:**
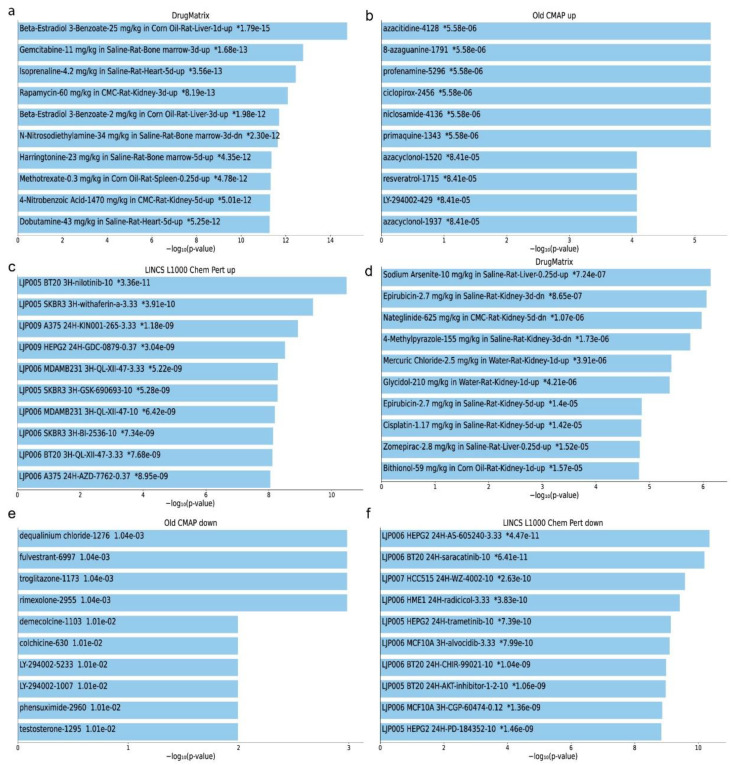
Top 10 enriched compounds that can be used as repurposing drugs against the top 250 co-upregulated (**a**–**c**) or down-regulated (**d**–**f**) genes in RCC according to DrugMatrix (**a**,**d**), cMap (**b**,**e**) and LINCS L1000 (**c**,**f**) databases. The significantly enriched terms of the associated compounds (*p* < 0.001) are denoted with an asterisk (*).

Similarly, the top compounds predicted to target the co-downregulated genes were as follows: “Sodium Arsenite”, “Epirubicin (3 days up)”, “Nateglinide”, “4-Methylpyrazole”, “Mercuric Chloride”, “Glycidol, Epirubicin (5 days up)”, “Cisplatin”, “Zomepirac” and “Bithionol” (DrugMatrix); “arecoline”, “trimethoprim”, “monorden”, “benzamil”, “gibberellic acid”, “testosterone”, “cantharidin”, “monocrotaline”, “eucatropine” and “ascorbic acid” (cMap); and “parthenolide”, “piplartine”, “ruxolitinib”, “AT-7519”, “thiostrepton”, “BRD-K08316444”, “np-009169”, “XMD11-85H”, “WZ-3105” and “niclosamide” (LINCS L1000) (Figure 11).

### 2.7. In Vitro Validation of Repurposed Drugs

We validated the effects of six repurposed drugs on the viability of HEK-293 cells. We selected BW-B70C, Lasalocid sodium, Catecholamine, Ifosfamide, Cyclosporin A and Etoposide, all of which were predicted to target co-upregulated genes in kidney cancer. Of these, five drugs were shown to affect HEK-293 viability (BW-B70C, Lasalocid sodium, Catecholamine, Cyclosporin A and Etoposide), whereas Ifosfamide did not show any effect in vitro.

**Figure 12:**
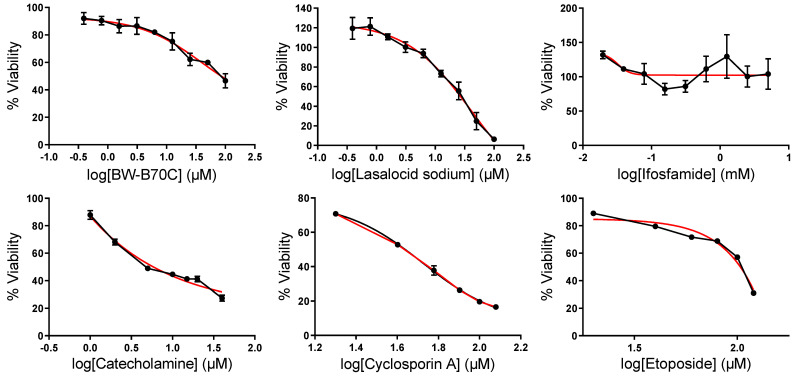
Curve fitting analysis to determine the effective inhibitory concentrations of BW-B70C, Lasalocid sodium, Ifosfamide, Catecholamine, Cyclosporin A and Etoposide on HEK-293 cells. The cytotoxicity effects of the drugs were measured using MTT assays (n = 3). To obtain IC50s of the drugs, an exponential two-phase decay model (marked as red line) was fitted to the dose-response curves (solid black lines) of single treatment of each drug on HEK-293 cells.

BW-B70C and Lasalocid sodium were shown to be effective against HEK-293 viability, with IC_50_ values of 42.30 μM (r^2^ = 0.8543) and 40.99 μM (r^2^ = 0.9368), respectively. When tested at higher concentrations, BW-B70C was found to reach a plateau of around 50% cytotoxicity. Ifosfamide was tested at concentrations up to 5 mM, but it was not found to have any cytotoxic effect against HEK-293 cells (IC_50_ = 0.032 μM, r^2^ = 0.1077). Interestingly, Catecholamine, Cyclosporin A and Etoposide were also shown to have a cytotoxic effect in HEK-293 cells, with IC_50_ values of 311.8 μM (r^2^ = 0.842), 52.98 μM (r^2^ = 0.987) and 2913 μM (r^2^ = 0.9621), respectively (Figure 12). The above results provide a strong validation to our drug-repurposing prediction methodology, which appears to be successful by a rate of 83.3% (five out of the six predicted drugs).

## 3. Discussion

Although significant progress has been made in understanding the complex molecular pathways that lead to kidney cancer development, there is still much more to learn. As a result, a great spectrum of genetic factors, perturbations and aberrations are to blame for the disease’s emergence and progression. Understanding pathways and conducting in-depth research on the genes involved in renal cancer necessitate a better understanding of their molecular signatures. In this study, we used a systems biology methodology to search for co-deregulated gene profiles, upstream regulators, networks and hub proteins across the different subtypes of RCC, as well as their protein–protein interactions. We identified commonly deregulated genes as well as upstream regulatory kinases and transcription factors that drive different patterns of gene expression in two groups: kidney cancers vs. normal tissue and kidney cancer cells or tissue with a single-gene perturbation.

Using the CD method, we initially discovered 161 co-upregulated genes (72 genes in RCC vs. normal and 89 genes in single-gene perturbation studies) and 97 co-downregulated genes (74 genes in RCC vs. normal and 23 genes in single-gene perturbation studies). We discovered the enriched pathways in which these co-DEGs participate. We then searched for compounds or drugs that can upregulate or downregulate these co-DEGs using three databases (DrugMatrix, cMap and LINCS L1000). In addition, we reconstructed the PPI networks to better understand the connections between co-DEGs and discovered several hub proteins that belong to either TFs or protein kinases and behave as signaling mediators.

Interestingly, among the top co-upregulated TFs in kidney cancer, we prioritized the MYC proto-oncogene, CCAAT Enhancer Binding Protein Data (CEBPD) and RELA proto-oncogene, NK-kB subunit (RELA). The MYC gene is frequently altered in >30% of human malignancies [10,11], spanning a wide spectrum of tumor subtypes. Especially in human renal cell carcinoma, MYC is frequently mutated or overexpressed. In specific, various gene amplifications, chromosomal translocations, viral integrations and regulatory mutations have been detected in its promoter or enhancer regions. Such changes have been linked to crucial stages of carcinogenesis, including its initiation, development and maintenance. Of note, the vast majority of the genomic rearrangements found at the MYC locus do not directly impact the MYC protein coding area. This is in line with the theory that MYC-driven malignancies are caused by dysregulation of MYC expression rather than changed or neomorphic changes in its protein function [10,11]. Unfortunately, the MYC oncoprotein is a difficult therapeutic target. The difficulty of defining a consistent set of target genes and developing safe MYC-specific therapeutics is due to the intricacy of its activity as a ubiquitous and promiscuous amplifier of gene expression [12].

CEBPD is a TF that belongs to the family of CCAAT/enhancer-binding proteins. Mainly, those TFs are related to biological processes, such as cell differentiation, growth arrest, cell death and motility [13]. Furthermore, CEBPD plays an important role in the regulation of genes associated with inflammatory responses and the activation and/or differentiation of macrophages. CEBPD has been linked to a variety of cancers, including colorectal [14], breast [15] and cervical cancer, and is frequently downregulated [16]. Because of its positive interactions with cyclin D1 and cyclin E, CEBPD appears to have tumor suppressive functions in breast cancer while being reduced or undetectable in invasive/metastatic cancer and tumor promoters [17]. Nonetheless, several studies investigate the use of CEBPD or its pathway as a cancer-fighting strategy. CEBPD upregulation via the p38/CREB pathway with HMDB as an activator was shown to activate PPPARG2 and chop GADD153 while attenuating E2F1, leading to the death of cancer cells [16]. Another way to use CEBPD against cancer is in combination with other compounds. Specifically, the combination of prodrug (PF)-CEBPD and PF-granzyme B showed a cumulative effect on the apoptosis pathway and raised apoptosis in PrCa cells. The prodrug (PF)-CEBPD was shown to increase the levels of procaspase-8, and the prodrug PF-granzyme B was shown to activate caspases 3 and 8 [18].

RELA is the most common member of the REL/Nuclear Factor-kB (NF-kB) family of dimeric transcription factors, which regulate the expression of several genes involved in differentiation, cell growth, regulation of apoptosis, neoplastic transformation and cytokine production [19]. RelA is up-regulated in colorectal cancer, where it seems to participate in tumor angiogenesis [20], but is has also been mentioned to be involved in other types of cancer as well [21]. In another study, Lehmann et al. found that RelA/p65 nuclear translocation as well as RelA/p65 DNA binding activity could be markedly diminished using HDAC inhibitors, which could thus be used to suppress NF-kB activity and lead to enhanced apoptosis and chemosensitization of pancreatic cancers [22].

From the list of co-downregulated genes in RCC, we highlight the TF zinc finger MIZ-type containing 1 (ZMIZ1) along with lysine acetyltransferase 2A (KAT2A) and Negative Elongation Factor Complex Member E (NELFE). ZMIZ1 belongs to the family of PIAS (protein inhibitor of activated STAT) proteins and controls the activity of other transcription factors (e.g., p53 and the androgen receptor Smad3/4). Even more, ZMIZ1 seems to be related to translocation involving chromosomes 9 and 10 in lymphoblastic leukemia. Another study examined the link between the expression of ZMIZ1 and Wilms tumor due to the fact that ZMIZ1 is associated with cancer etiology with unknown specific function in tumor development and progression. Recently, ZMIMZ1 knockdown was shown to affect invasion and metastasis of Wilms tumor by regulating the Notch signaling pathway, thus it could be used as a new promising therapeutic strategy for the disease [23]. Furtehrmore, using chromatin immunoprecipitation assays (ChIP), Li et al. showed that ZMIZ1 recruits ARQ9 on the promoter of the prostate specific antigen (PSA) gene, modulating the polyQ tract length of AR in prostate cancer cells [24]. In addition, ZMIZ1 is used as a prognostic marker of renal cell cancer, among other types of cancers [25], rendering it a possible target for anticancer drugs.

KAT2A histone acetyltransferase is a transcriptional activator, but it can also repress NK-κB through ubiquitination of its RELA subunit. KAT2A has been reported to promote carcinogenesis in colorectal cancer by interacting with RNA-binding protein PTBP1 [26] and to modulate cGAS through increasing expression and post-translational modification in systemic lupus erythematosus [27]. In addtrion, KAT2A-mediated histone succinylation was recently found to play a new role in the regulation of gene expression and β-catenin stability to promote tumor cell proliferation and invasion of pancreatic carcinoma cells [28].

The transcription factor Negative Elongation Factor (NELF) is part of a complex that represses transcript elongation by RNA polymerase II. It is similar to nuclear RNA-binding proteins, however, it is unknown whether it binds to RNA. NELF protein contains a tract of alternating arginine (R) and aspartic acid (D) residues. NELF, among others is involved in pancreatic cancer metastasis and gastric cancer progression [29]. Even if evidence shows that NELFE is important in tumorigenesis, the actual relation between gastric cancer and NELFE remains unclear. A study highlight, that NELFE-Wnt/β-catenin-CSNK2B hubs can be new candidate targets in gastric cancer through their positive connection, as they found that all these hubs are upregulated [30].

Similar to the category of co-upregulated genes in RCC, MYC, Krupple Like Factor 4 (KLF4) and NELFE were also found among the top up-regulated TFs among the single-gene perturbation experiments. KLF4 is another transcription factor whose main purpose is to regulate cellular processes such as cell growth, proliferation and differentiation. It has been studied intensely since it is one of four factors that are obligatory for the induction of pluripotent stem cells [31]. KLF4 is also involved with β-catenin. Precisely, KLF4 interplays with the transcription factor Sox9, and both antagonize β-catenin and inhibit TCF activity in cancer cells [32], rendering KLF4 as another candidate therapeutic target against cancer.

NELFE and MYC were also listed within the down-regulated genes in single-gene perturbation experiments. In this category, we also highlighted the TF Tumor Protein P63 (TP63), a transcription factor that mainly acts as a sequence-specific DNA binding transcriptional activator or repressor. TP63 can induce the expression of various proteins using different promoters or splicing, DNA-binding or functioning through their transactivation in squamous cell carcinoma (SCC), lung cancer and other cancer types [33,34]. TP63 is part of the TP63/Six2-CCat1-EGFR cascade, which is activated by the SCC-specific DNA/RNA protein complex [35]. In addition, TP63 was found to bind to transcriptional regulatory regions of Ataxia-telangiectasia group D complementing gene (ATCD), inducing its expression in aggressive basal bladder cancers [36]. These findings support our findings that TP63 is another interesting target for treatment in kidney cancer.

Furthermore, among the co-DEGs acting as protein kinases, we highlighted the hubs Casein Kinase 2 alpha 1 (CSNK2A1), Mitogen-activated protein kinase 14 (MAPK14) and Mitogen-activated protein kinase 1 (MAPK1). In specific, CSNK2A1 is a protein kinase that phosphorylates various proteins, including sirtuins (SIRT1), and relates to a number of cellular processes, including apoptosis, circadian rhythm and cell cycle control. CSNK2A1 has been previoulsy reported to be a predictive biomarker and a therapeutic target in different types of cancer [37]. Bae et al. showed that CSNK2A1 and SIRT6 are indicators of poor prognosis in breast cancer and that CSNK2A1-mediated phosphorylation of SIRT6 could be involved in the progression of breast cancer [38]. Similarly, CSNK2A1 has been reported as a protein with a positive role in gastric cancer and as a possible therapeutic target. Protein β-1,3-galactosyltransferase5-AS1 (B3GALT5-AS1) links to CSNK2A1 and regulates its expression. In an experiment with B3GALT5-AS1 knockdown cancer cell viability, both invasion and migration were reduced and cell death was promoted. Once CSNK2A1 was overexpressed, all the above were partially reversed, showing that CSNK2A1 is another probable drug target in cancer [39].

MAPK14 is a protein kinase that belongs to the large family of MAP kinases. Similar to others kinases, it is related to a large variety of cellular processes, including differentiation, proliferation, development and transcription regulation. MAPK14 acts as an integration point for several biochemicals signals. It is mainly triggered by proinflammatory cytokines and different environmental stresses, and it is activated with phosphorylation or autophosporylation. MAPK14 has been described either as a cancer inducer or suppressor in different studies. In a study by Liu et al. [40], the proliferation and migration of ccRCC cells was repressed upon MAPK14 knockdown and then incompletely reversed when Cell division cycle 25B (CDC25B) was overexpressed. The study recommended the downregulation of both MAPK14 and P-MAPK14 by downregulating CDC25B as a possible method to suppress the proliferation and migration of ccRCC [40]. By downregulating MAPK14 in renal cancer cells lines using a 2-Furanone compound called “compound 3a”, Abd El-Hameed et al. [41] showed that these have promising anticancer activities and corroborrate our suggestion that MAPK14 could be a very promising target against kidney cancer.

In addition, MAPK1 is another protein kinase belonging to the extracellular signal-regulated kinase (ERKs) family. Similar to MAPK14, MAPK1 needs phosphorylation from other upstream kinases for its activation and is related to a variety of cellular processes. After its activation, it translocates into the nucleus of stimulated cells, where it phosphorylates various nuclear targets, including Aurora-A kinase gene (AURKA) [42]. Fei et al. [43] showed that miR-378 inhibits cell proliferation, cell cycle progression and cell migration and invasion and induces cell apoptosis in gastric cancer via targeting MAPK1. In addition, silencing MAPK1 using siRNA significantly inhibits the invasion and metastasis of cervical cancer, in which MAPK1 expression is particularly high [44]. The above findings suggest that MAPK1 targeting could be used against kidney cancer as well.

The hubs CSNK2A1, Cyclin-dependent kinase 4 (CDK4) and Cyclin-dependent kinase 1 (CDK1) were identified as the most relevant kinase proteins among the up-regulated genes with a single-gene perturbation. CDK4 belongs to the Ser/Thr protein kinase family and acts in the G1-S phase of the cell cycle, where it phosphorylates the retinoblastoma gene (RB) product. Importantly, various mutations in this kinase are associated with cancer. INK4 family members bind CDK4 and inhibit its kinase activity. In kidney cancer patients, Sun et al. [45] showed that the INK4 family of genes is upregulated, positively affecting the prognosis of kidney cancer and inhibiting tumor development. Additionally, Ara-c promotes the upregulation of INK4 family genes and regulates the cell cycle-dependent genes CDK4 and cyclin D1 (CCND1) independent of the INK4 family genes [45]. CDK4 and other cell cycle regulation proteins could be attenuated by the nuclear receptor peroxisome proliferator-activated receptor α (PPARα) antagonist [46]. CDK4 is a very promising therapeutical target, but there is much more investigation needed with present therapeutics counting immune checkpoint inhibitors [47].

Similar to CDK4, CDK1 belongs to the Ser/Thr protein kinase family and is a catalytic subunit of M-phase promoting factor (MPF) complex. The latter is important for the transition of the eukaryotic cell cycle from the G1 to the S phase and from the G2 phase to mitosis. For its activation, CDK1 binds to B-type cyclins. The expression of CDK1 and related genes could efficiency be used as part of a prognostication scoring system in combination with other genes for renal cancer. Genes of the prognostication scoring system were deregulated and, specifically, CDK1 expression was notably low [48]. A study by Pan et al. [49] showed that CDK1 expression could be managed by the eukaryotic initiation factor 3d (EIF3D), the knockdown of which paused cell proliferation due to down-regulation of Cyclin B and CDK1. Another study showed that miR-31-5p acts as a tumor suppressor in kidney cancer by targeting CDK1 and constraining proliferation, migration, invasion and cell cycle [50]. The above shows that CDK1 is already a possible drug target in kidney cancer, and our findings corroborate this statement.

We finally highlight the hubs Homeodomain Interacting Protein Kinase 2 (HIPK2) and Extracellular-signal regulated kinase (ERK1) among the most connected protein kinases in the co-downregulated genes in single-gene perturbation studies. HIPK2 is part of the homeodomain-interacting protein kinase family and acts as another serine/threonine kinase that interrelates mainly with homeodomain transcription factors and could be either a co-repressor or co-activator contingent on the transcription factor. HIPK2 has been reported to be overexpressed in cases of cervical cancer. Furtermore, the expression of HIPK2 kinase was meaningfully lower in cases of cervical cancer in stage I than cases of cervical cancer in stage II or III, showing a connection between HIPK2 and cancer development [51]. HIPK2 can regulate the expression of P53, which is related to cellular stress responses caused by cancer development. The action of P53 is mainly dependent on post-translational alternations, which start with DNA damage. HIPK2 phosphorylates P53 and controls its apoptotic function. Therefore, the interaction across the above-mentioned hubs suggests that they could be used as a remarkable target for cancer treatment [52].

ERK1 is a protein-serine/threonine kinase connected with ERK2 protein. They both contribute to the RAS/RAF/MEK/ERK cascade, which adjusts a number of cell processes, such as cell cycle progression, cell survival, cell adhesion, cell proliferation, cell differentiation and others. ERK1/2 is able to phosphorylate a large number of substrates, cytoplasmic and nuclear, such as transcription factors and regulatory molecules. An important notice is the high activity of the RAS-MEK-ERK cascade in one-third of all human tumor types [53]. Therefore, ERK1/2 inhibitors are in the spotlight for their anticancer effects. A number of ERK inhibitors are examined with clinical trials for the operation of cancer, and, in some cases, they showed remarkable anticancer actions [54]. In a study in oral cancer, a connection between ERK1/2 and CD44 was confirmed. In particular, the authors identified CD44 as an important target of ERK1/2 in the promotion of tumor aggressiveness [55]. Another study by Mendes et al. [56] used an MEK inhibitor (PD98059) able to block the phosphorylation by ERK1/2 kinase, resulting in the reduction of MMP2 expression in breast cancer cells. These findings corroborate our hypothesis that ERK1 can also be targeted in kidney cancer.

Furthermore, we predicted potential repurposing drugs that could target the above-mentioned co-DEGs or hubs using in silico analysis of data from three different databases (DrugMatrix, cMap and LINCS L1000). Importantly, the cytotoxic effects of BW-B70C, Lasalocid sodium, Catecholamine, Cyclosporin A and Etoposide were validated in vitro using the HEK-293 cell line, whereas Ifosfamide did not show any effect on the viability of the cells. Among these drugs, we discuss below Etoposide (DrugMatrix), Haloperidol (cMap down) and BW-B70C (LINCS L1000 Chem Pert down), which were predicted to down-regulate the co-upregulated genes in kidney cancer. Of these, Etoposide and BW-B70C were also proven to reduce HEK-203 cellular viability. We further discuss Triamterene (DrugMatrix), Chlorphenesin (cMap down) and BRD-K79459005 (LINCS L1000 Chem Pert down), which were predicted to induce the co-downregulated genes.

The advantage of HEK293 cells that we used is that they are very easy to grow and to maintain, with high reproducibility, which makes them preferable over other less-robust and slow-growing cell lines. In addition, they are very efficient at protein production and accessible for transfection. They have also been widely used to analyze the effect of drugs on sodium channels and the interactions between different proteins and to produce proteins and drugs. Their disadvantage is that, if extensively cultured, their growth rate and translation efficiency is affected, although high passages increase their tumorigenicity. In addition, they display some cytogenetic instability.

Etoposide is already used as a treatment against testicular and small cell lung tumors. This compound works as an inhibitor of DNA synthesis by forming a complex with DNA and topoisomerase II and inhibiting repair from the latter. As a result, cell death is promoted due to the large number of DNA breaks and the suspension of cell division [57,58]. A study for the improvement of overall survival and event-free survival for clear-cell sarcoma of the kidney (CCSK) patients examined the use of Etoposide and cyclophosphamide as treatment against this cancer type. The outcomes of the study demonstrated better consequences only for CCSK patients with stage I and II of the disease. Patients with higher stages did not show better results in comparation with the previous treatment [59]. Interestingly, our findings support that Etoposide has a cytotoxic effect on HEK-293 cells and could thus be used for therapeutic purposes against kidney cancer.

Haloperidol is mainly used as a treatment for schizophrenia and other psychoses. It is commonly used to reduce nausea and vomiting in patients with tumors without any harmful actions on patients’ health [60,61]. Supporting our findings, new studies displayed that the compound has anticancer properties, such as the inhibition of growth and proliferative activities on cancer cell lines from various types, including prostate and breast cancer [62,63]. Either way, all the studies underline the importace of further investigation about the use of Haloperidol as an anticancer treatment in kindey cancer.

BW-B70C is a selective inhibitor of arachidonic acid 5-lipoxygenase [64]. Interestingly, this compound has not been investigated intensely as a possible drug against kidney cancer. Corroborating our notion that this is a putative drug in kidney cancer, Villegas et al. [65] showed that the lipoxygenase inhibitor BW-B70C selectively killed human leukemic cells by dampening the NOTCH1-PI3K/AKT-eNOS axis. Here, we showed that BW-B70C indeed has strong cytotoxic effects on HEK-293 cells and could therefore be used as a treatment modality against kidney cancer.

Triamterene is a compound usually used as potassium-sparing diuretic, and its main purpose is to control hypertension [66]. It has previoulsy been examined as an anticancer treatment in colon cancer cell lines HCT116 and CT26, in which it had low cytotoxicity and quite promising results [67]. Nevertheless, more studies are warranted on the possible use of triamterene against lung cancer.

Chlorphenesin is a compound being used in different pharmaceutical industry sectors. Two compounds are known by the name Chlorphenesin, the first is often being used in the cosmetic industry and the other, known as Chlorphenesin carbamate, is frequently being used for muscle relaxation [68]. Other uses of Chlorphenesin are against fungi and bacteria, especially in vaginal and dermal infections. It looks to be effective against dermal squamous cell carcinoma, and, therefore, perhaps it could also be used to treat kidney cancer. Its mode of action involves the induction of cell-mediated immune responses of the host and does not appear to have cell toxicity [69].

BRD-K79459005 is a lactam and azamacrocyclen. Unfortunately, there is no remarkable information on this compound as an anticancer drug.

Beta-Estradiol 3-benzoate (also known as Estradiol benzoate) is a derivative from the sex hormone estradiol. Mainly, it is branded for its capacity as a blood coagulability inducer. Estradiol benzoate has been used therapeutically against breast cancer in combination with other compounds, and some patients exhibited improvement for a short period after treatment. In any case, the results are encouraging, but still further examination is needed for the use of Estradiol benzoate as part of treatment against kidney cancer.

Dequalinium chloride is a compound with frequent use as an antiseptic drug, but also as an antineoplastic and antifungal agent. It is is an inhibitor of XIAP and can also act as a mitochondrial targeting agent, which renders it as a promising therapeutic agent against malignant glioma [70]. Therefore, this compound could also be used as a possible target against kidney cancer, but exhaustive further investigation is still needed. A study observed the toxicity of Dequalinium chloride and suggested a maximum dose of the compound that did not cause toxic effects and mortality [71].

AS-605240 is an active inhibitor of PI3Kγ. By blocking the PI3Kγ kinase through phosphorylation, the tumor shrinks. An additional benefit of the of the phosphorylation of PI3Kγ kinase is the obviation of anthracyclines cardiotoxicity [72]. This particular ability of AS-605240 to be selective for the PIK3γ kinase [73] renders it a very promising drug.

Sodium arsenite, among others, is an antineoplastic agent. Its derivative, sodium metaarsenite, is a possible anticancer compound due to its ability to stop the action of telomerase, a critical enzyme with high activity in the majority of cancer cells. The inhibition of the telomerase results in the induction of cancer cell death and the inhibition of cancer cell development. Nevertheless, more studies on this compound are required, especially in kidney cancer.

Arecoline is an alkaloid from the betel nut (Areca catechu), the fruit of palm trees. In line with our findings demonstrating that Arecoline could be used as a drug against kidney cancer, previous studies also show that this compound acts as a tumor inhibitor. In specific, this compound is known to act as an inhibitor of p53 and a repressor of DNA repair [74,75].

Finally, Parthenolide is a sesquiterpene lactone that is found in Tanacetum and has anticancer chemotherapeutic, anti-metastatic, anti-angiogenic, anti-inflammatory and antinociceptive properties. Parthenolide acts as a partial agonist and desensitizes transient receptor potential ankyrin 1 (TRPA1) channels, preventing the release of calcitonin gene-related peptide (CGRP). Parthenolide also impedes the ATPase activity of NLRP3 and the protease activity of caspase 1. Parthenolide inhibits cell migration and tubule formation by decreasing NF-B, VEGF and IL-6 expression and increasing IB kinase expression in multiple myeloma cells. Parthenolide reduces MCL-1 levels while increasing MAIP-1 levels in non-small cell lung cancer (NSCLC) cells, causing ER stress and inducing cell cycle arrest and apoptosis. Parthenolide activates NADPH oxidase and increases ROS generation in breast cancer cells, increasing p-JNK levels while decreasing NF-κB, VEGF and MMP2/9. In vivo, parthenolide inhibits the growth and metastasis of tumors. In addition, this compound has been identified as an agonist of adiponectin receptor 2 [76]. Recently, Parthenolide was studied as a therapeutic agent against kidney cancer with positive indications [77], making it a compound that could be considered as a good target for the treatment of kidney cancer.

## 4. Materials and Methods

### 4.1. Extraction and Filtering of Gene Expression Signatures from the GEO Database

We gathered data from 10 different studies and divided them into two groups: (1) studies focusing on gene expression differences between renal cancer and adjacent normal tissue (4 GEO studies) and (2) studies focusing on a single-gene perturbation (e.g., mutation, knock-out, knock-in, etc.) vs. wild-type cells or tissues (6 studies). Gene expression signatures were extracted using GEO2Enrichr [78]. We began by looking at studies involving human and mouse cell lines or tissue samples. Major information such as gene, drug and disease name were automatically filled in submission forms from authorized websites: HUGO Gene Nomenclature Committee for gene names, DrugBank for drug names and Disease Ontology for disease names.

The GEO datasets that we selected for each category were as follows: GDS507, containing 8 normal samples (4 normal human kidney and 4 normal cells from ccRCC patients) and 9 renal cell carcinoma samples; GDS1344, containing 22 molecular class type 1 (described by excellent survival) samples (14 histologic class 1, 5 histological class 1 and 2A and 3 histological class 2A) and 13 molecular class type 2 (described by poor survival) samples (1 histologic class 2A and 11 histological class 2B); GDS2881, having 10 wild-type samples and 10 ccRCCs (5 stage I and 5 stage II ccRCCs); GDS3274, containing 9 oncocytomas and 9 chroRCCs; GDS1748, with 4 controls (2 E14.5 and 2 E18.5) and 4 Lim1 null mutant samples (2 E14.5 and 2 E18.5), where the terms E14.5 and E18.5 described the embryonic day; GDS2010, with 2 control and 2 WTAP knockdown samples; GDS4282, having 22 normal cortex BAP1 wild type/PBRM1 wild type and 57 gene perturbation samples (ccRCC 4 BAP1 loss/PBRM1 wild type 12 BAP1 wild type/PBRM1 wild type, tumorgraft_p0 2 BAP1 loss). We looked at four different GEO datasets to see which co-DEGs were present in kidney cancer tissues compared to their normal tissue. The co-upregulated genes in this category were substantially enriched tumograft_p1 2 BAP1 wild type/PBRM1 loss 1 BAP1 wild type/PBRM1 wild type, tumorgraft_p2 2 BAP1 wild type/PPBRM1 loss, 1 BAP1 wild type/PBRM1 wild type, tumorgraft_p3 BAP1 wild type PBRM1 wild type, BAP loss/PBRM1 wild type, tumorgraft_p4 2 BAP 1 loss/PBRM1 wild type, 1 BAP1 wild type/PBRM1 wild type, tumorgraft_p7 1 BAP1 wild type/PBRM1 wild type, tymorgraft_p8 2 BAP1 loss/PBRM1 wild type, tumorgraft_p9 1 BAP1 wild type/PBRM1 wild type and tumorgraft_p1_from metastasis 1 BAP1 wild type/PBRM1 loss; GDS4802, containing two control samples and two WTX overexpression samples; GDS4864, with three control samples, four VHL knockout samples, five induced acute kidney injury and four VHL knockout + induced acute kidney injury; and GDS5415, having four controls (one sample at the age of five weeks, three samples at the age of four months) and four Lin28-derived tumors (one sample at the age of five weeks, three sample at the age of four months).

We re-processed the extracted expression signatures to filter their quality and check data integrity, as previously explained [79]. In detail, different steps of quality control filters were applied to improve the extracted GEO signatures. We first performed integrity checks using the association between GEO studies (GSE or GDS) and samples within these studies (GSMs) by re-processing all the collected gene expression signatures. Signatures in which GSMs did not match their GSE or GDS, as well as signatures with the same GSMs in the control and perturbation groups, were automatically detected and removed. The next filter was applied only to the single-gene perturbation collection. We checked whether the gene symbols used are valid HGNC gene symbols, removing all entries with invalid genes. The next filter was semi-automatic: we corrected signatures in which the control and perturbation samples were switched. Our final filter was to manually check if the submitted signatures agree with the descriptions associated with the original GEO studies.

We also checked for batch effects across the different GEO studies and improved the quality of the gene expression signatures. We first obtained the “scan date” from the raw microarray CEL files and assumed that the experiments were performed on the same dates that were listed within the experimental batch. We then quantified the batch effect using principal variation component analysis (PCVA), which attributes the variation in the gene expression data to known sources such as batches and experimental conditions. Batch effects were corrected using the surrogate variable analysis (SVA) algorithm with default parameters [80].

### 4.2. Differential Gene Expression and the Co-Deregulated Genes across Different Studies

We used the Characteristic Direction (CD) algorithm [8] to calculate the differential gene expression. GEO2Enrichr was applied to examine under-expressed and over-expressed gene sets (termed “down” and “up”, respectively) in renal cancer with the adjacent normal tissue. For single-gene perturbation experiments, the deregulated expression was considered against the wild-type cells/tissue. We set a cut-off of 250 genes for all the DEGs and excluded all the deregulated genes that were not detected in at least 2 independent studies.

### 4.3. Upstream Regulators of the Co-Deregulated Genes and Protein–Protein (PPI) Interactions

The Expression2Kinases (X2K) method was used to deduce the upstream regulatory networks from a list of co-DEG signatures in the two categories on which we concentrated our efforts (cancer vs. normal and single-gene perturbation vs. wild type). Using Enrichr, we inferred the gene networks that are anticipated to govern the expression of the co-DEGs by combining transcription factors (TF) enrichment analysis, protein–protein interaction (PPI) network expansion and kinase enrichment analysis (KEA) [81].

The PPI networks that were created included TFs, protein kinases and intermediate proteins for each class of co-DEGs. Using X2K (https://maayanlab.cloud/X2K/; 1 January 2021), we received enrichment lists of TFs by recognizing proteins that interact with them. We also presented a ball-and-stick diagram for each category as a subnetwork of the TFs and their interacting proteins.

The sizes of the nodes in the network were proportional to their degree. In the PPI expansion network, all the nodes were marked with k-core values. The proteins with higher k-core values (hubs) are more centralized in the network and have a stronger capacity of modulating adjacent genes.

### 4.4. Gene Ontology (GO) Enrichment and Kyoto Encyclopedia of Genes and Genomes (KEGG) Pathway Analysis

The biological properties, molecular function and cellular components of the top co-DEGs were investigated using GO and KEGG pathway enrichment analysis. The hypergeometric test was employed to uncover GO terms that were considerably enriched when compared to the entire human genome. The Fisher’s exact test was used to determine statistical significance. Benjamini–Hochberg (BH) was used to correct *p*-values, and an adjusted *p*-value (adj-*p*) of 0.05 was selected as threshold of statistical significance. Uniform Manifold Approximation and Projection (UMAP) was used to cluster similar gene sets together. Clusters were computed using the Leiden algorithm [82]. Points were plotted on the first two UMAP dimensions. Figures were created using BokehJS 2.3.2.

### 4.5. Validation of the Co-Deregulated Gene Signatures in the TCGA and across Different Molecular and Immune Subtypes in Kidney Cancer

We validated the calculated signatures in kidney cancer after extracting the read counts of RNA-seq data from the TCGA-KIRC (523 tumor samples and 72 controls), TCGA-KIRP (286 tumor samples and 32 controls) and TCGA-KICH (66 tumor samples and 25 controls) datasets using the Genomic Data Commons data portal (https://portal.gdc.cancer.gov/; 15 May 2022). We then normalized the read counts to log_2_(TPM + 1) values, as previously described [83]. The expression of the “UP genes” signature, which was composed of 72 genes, and of the “DOWN genes” signature, composed of 74 genes, were explored with limma [84], using a threshold of significance of log_2_FC = 1 and q-value = 0.01. The UP and DOWN gene signatures were explored across KICH, KIRC and KIRP, as well as across four mRNA clusters in KIRC (cluster 1, n = 145; cluster 2, n = 88; cluster 3, n = 93; cluster 4, n = 85).

In addition, we explored the expression of the top co-deregulated genes in kidney cancer across different molecular subtypes in kidney renal papillary cell carcinoma (KIRP) (C1, C2a, C2B, C2c-CIMP) and immune subtypes in KIRP, kidney chromophobe (KICH) and kidney renal clear-cell carcinoma (KIRC) (C1, wound healing; C2, IFN-γ-dominant; C3, inflammatory; C4, lymphocyte-depleted; C5, immunologically quiet; C6, TGF-b-dominant), as previously described by Thorsson et al. [85].

### 4.6. Gene Set Analysis in Kidney Cancers

Gene Set Cancer Analysis (GSCA) was used to investigate and explore the gene set of the hub proteins that we identified above across kidney cancers (also per stage) in the TCGA dataset. We validated the mRNA expression profiles of the key hub genes across different stages in KICH, KIRC and KIRP tumors and explored the pathway activity between high and low mRNA expression of these genes in kidney cancer. In addition, gene set variation analysis (GSVA) scores were calculated for the designated hub genes in KICH, KIRC and KIRP tumors [86].

### 4.7. Detection of Repurposing Drugs in Kidney Cancer

DrugMatrix is the world’s largest molecular toxicology reference database and informatics system, containing the comprehensive results of thousands of highly controlled and standardized toxicological experiments in which rats or primary rat hepatocytes were systematically treated with >600 therapeutic, industrial or environmental chemicals at both non-toxic and toxic doses. It contains various gene expression datasets created by extracting RNA from the toxicologically relevant organs and tissues, the analysis of which is performed with the GE Codelink rat array and the Affymetrix whole genome 230 2.0 rat GeneChip array (ntp.niehs.nih.gov/drugmatrix/; 1 April 2021).

According to the signatures that we found, the Connectivity Map (CMap, https://clue.io/cmap; 15 June 2021 [87]) was employed to find repurposing drugs that might potentially induce or reverse the deregulated gene expression patterns in kidney cancer. “Connected” perturbations elicited substantially comparable, or different, expression patterns. A positive connectivity value (closer to +1) implied that the medications may cause kidney cancer cells to become malignant, whereas a negative connectivity value (closer to −1) indicated that increased similarity between the genes and the pharmaceuticals may cause kidney cancer cells to revert their state. Using the hypergeometric probability test, drugs were statistically linked to the condition.

The LINCS L1000 project collects gene expression profiles for thousands of perturbagens at a variety of time points, doses and cell lines. The LINCS L1000 Chem Pert (Chemically Perturbed) library was used to detect repurposing drugs for each gene set (https://lincsproject.org/, 15 June 2021) [88].

### 4.8. Cell Culture

HEK-293 cells were routinely maintained in Dulbecco’s Modified Eagle’s medium (DMEM/Ham’s F-12 with L-Glutamine) supplemented with 10% heat-inactivated fetal bovine serum (FBS) and 1% antibiotic cocktail mix. The cells were grown to confluence in 25 cm^3^ or 75 cm^3^ cell culture flasks loosely capped (Greiner Bio-One Ltd., Stonehouse, Gloucestershire, UK) at 37 °C in 5% CO_2_ and 95% humidity. The flasks were left to reach adequate confluence before conducting each experiment.

### 4.9. MTT Assay

The MTT assay was conducted as described previously [89]. Briefly, 10 × 10^3^ cells were seeded in normal medium in a 96-well plate and left to attach overnight. The cells were then treated with increasing concentrations of the drugs (BW-B70C, Lasalocid sodium, Ifosfamide, Catecholamine, Cyclosporin A and Etoposide) or vehicle for 48 h. Then, 15 μL of MTT solution (5 mg/mL in PBS) was added for 4 h, the medium was removed, and 100 μL of DMSO was used to completely dissolve the purple formazan crystals. The absorbance of the test plate was read at 570 nm. The absorbance for each compound was subsequently normalized to the vehicle-treated cells prior to plotting. Data were statistically analyzed using non-linear regression (curve fit) to calculate the half-maximal inhibitory concentration (IC_50_ value) of each drug against HEK-293 cells in GraphPad Prism (v8.4.2).

## 5. Conclusions

Overall, we used a systems biology approach to investigate the co-deregulated genes in studies with kidney cancer or in studies that used single-gene perturbations in kidney cancer cells. We identified the important hub genes in each group acting as transcriptional regulators for the co-deregulated genes (transcription factors and kinases), and we examined the molecular networks generated by these co-DEGs. Apart from identifying the key genes involved in kidney cancers, we further proposed five repurposed drugs (BW-B70C, Lasalocid sodium, Catecholamine, Cyclosporin A and Etoposide), which could be used as a potential treatment against the disease. Overall, our findings validate previously published results and reveal the dynamic processes that take place during kidney carcinogenesis.

## Data Availability

The figshare repository was used to store the supporting data of our findings (doi:10.6084/m9.figshare.20756809).

## Supplementary Materials

The supporting information can be downloaded at: https://www.mdpi.com/article/10.3390/ijms24076577/s1.
